# Immune Regulation in Time and Space: The Role of Local- and Long-Range Genomic Interactions in Regulating Immune Responses

**DOI:** 10.3389/fimmu.2021.662565

**Published:** 2021-05-11

**Authors:** Liam P. Devenish, Musa M. Mhlanga, Yutaka Negishi

**Affiliations:** ^1^ Division of Chemical, Systems, and Synthetic Biology, Department of Integrative Biomedical Sciences, Institute of Infectious Disease & Molecular Medicine, Faculty of Health Sciences, University of Cape Town, Cape Town, South Africa; ^2^ Radboud Institute for Molecular Life Sciences (RIMLS), Radboud University Medical Center, Nijmegen, Netherlands; ^3^ Epigenomics & Single Cell Biophysics Group, Department of Cell Biology, Radboud University, Nijmegen, Netherlands; ^4^ Department of Human Genetics, Radboud University Medical Center, Nijmegen, Netherlands

**Keywords:** genome structure, immune regulation, rapid response, heterogeneity, autoimmune diseases

## Abstract

Mammals face and overcome an onslaught of endogenous and exogenous challenges in order to survive. Typical immune cells and barrier cells, such as epithelia, must respond rapidly and effectively to encountered pathogens and aberrant cells to prevent invasion and eliminate pathogenic species before they become overgrown and cause harm. On the other hand, inappropriate initiation and failed termination of immune cell effector function in the absence of pathogens or aberrant tissue gives rise to a number of chronic, auto-immune, and neoplastic diseases. Therefore, the fine control of immune effector functions to provide for a rapid, robust response to challenge is essential. Importantly, immune cells are heterogeneous due to various factors relating to cytokine exposure and cell-cell interaction. For instance, tissue-resident macrophages and T cells are phenotypically, transcriptionally, and functionally distinct from their circulating counterparts. Indeed, even the same cell types in the same environment show distinct transcription patterns at the single cell level due to cellular noise, despite being robust in concert. Additionally, immune cells must remain quiescent in a naive state to avoid autoimmunity or chronic inflammatory states but must respond robustly upon activation regardless of their microenvironment or cellular noise. In recent years, accruing evidence from next-generation sequencing, chromatin capture techniques, and high-resolution imaging has shown that local- and long-range genome architecture plays an important role in coordinating rapid and robust transcriptional responses. Here, we discuss the local- and long-range genome architecture of immune cells and the resultant changes upon pathogen or antigen exposure. Furthermore, we argue that genome structures contribute functionally to rapid and robust responses under noisy and distinct cellular environments and propose a model to explain this phenomenon.

## Introduction

Survival requires that organisms withstand the constant assault of external and internal threats. The most fundamental of these is coexistence with potential pathogens with the means to invade and cause harm in myriad ways. Consequently, mammals have evolved a complex network of immune responses. In addition to monitoring and responding to incursions, surveilling the patterns of normal tissue development and function is an essential immune process. Given the unrelenting burden this places on the organism, it follows that the immune cells responsible have developed, what is in many ways, a unique phenotype and regulatory state. Namely, immune cells must recognize and respond to infection immediately or run the risk of being overwhelmed by any number of invasive microbial species. The spectrum of potential pathogens is vast, ever-changing, and their virulence factors and means of invasion and evasion almost boundless. A diverse pool of dangers necessitates an equally diverse responsive potential. Fostering phenotypic and functional diversity is thus an indispensable feature of immune cells. This versatility includes a number of requirements: firstly, discretion - overlooking harmless features of internal and external environments thus preventing (or attenuating) unnecessary immune responses are essential in avoiding immune-mediated conditions. Secondly, immune cells must be poised for a rapid response replete with the functional specificity required for resolution. Here then, we have two salient features of the immune response: heterogeneity, and inductive capacity which is programmable. Without the successful synergy of these factors homeostasis is lost: the organism becomes victim to invasive pathogens or, alternatively, fails to contain internal dysregulation. These features are, therefore, crucial to the functioning of immune cells.

The function of immune cells is regulated through gene expression of immune genes. Gene expression patterns for inducible genes are contingent on developmental stage and environmental change. The process is complex and variegated; comprising multiple individual sequences usually in a predetermined order: Chromatin remodeling, transcription, post-transcriptional modification, translation, post-translational modification, and transportation. Regulation can thus occur at every step. Nucleosome remodeling at promoter regions prior to transcription, for example, is well-described (1). Nucleosomes at transcription start sites are depleted allowing RNA polymerase engagement by ATP-dependent chromatin remodeling agents, such as the SWI/SNF complex (1). The second step is the recruitment of general transcription factors and formation of the pre-initiation complex (PIC) (2). RNA polymerase II (RNAPII) within the PIC initiates transcription by synthesizing short RNAs, approximately 25-60nt in length, before the transcription complex pauses, awaiting further positive signaling. At this stage, the negative elongation factor (NELF) binds RNAPII together with DRB Sensitivity Inducing Factor to temporarily inhibit RNAPII reading (2). This pause is followed by elongation. P-TEFb is recruited to the PIC and, in turn, phosphorylates serine residues at the second and fifth amino acid from N-terminal; phosphorylation dissociates NELF allowing elongation to begin and the full-length transcript is subsequently synthesized (3, 4).

These are the minimum requirements for transcription; without additional potentiating factors baseline expression remains low. Synthesizing the number of transcripts of immune genes required following immune challenge necessitates the accruement of other activating elements to induce efficient transcription. Specific transcription factors (TFs) are one such example. Key to immune cells specifically is NFkB - known to induce most inflammatory genes while suppressing a cohort of metabolic genes. In the nucleus, NFkB regulates gene expression by recruitment of P-TEFb. Interestingly, NFkB also has both potentiating and inhibitory effects on gene expression through the variable recruitment of histone acetyltransferases and histone deacetylases (5–7).

The nucleosome is a central feature of DNA arrangement allowing for both compaction and functional control of genes. A strand of DNA makes 1.68 turns around a histone protein octamer, forming a fiber-like structure 11 nm in width. Post-translational modifications of these histones are well-entrenched regulators of this packing process. A variety of local histone modifications are associated with actively transcribed promoters: H3K4me3 recruits RNAPII to the promoter while H3K27ac recruits BRD4 which in turn recruits elongation factor P-TEFb (8–10). Additionally, p300, a histone acetyltransferase which uses acetyl-CoA as a substrate, performs histone crotonylation in macrophages. Although the mechanism remains unknown, crotonylation is associated with a greater degree of gene expression potentiation compared to acetylation (11).

In addition to local genomic structures, distal elements such as enhancers are indispensable in supporting efficient gene expression. Active enhancers are identified by association of H3K4me1 and H3K27ac and a specialized family of RNAs, enhancer RNAs (eRNAs) which are transcribed from these sites (12). Although the mechanism of enhancer action - for example, the necessity of eRNAs - remains controversial, it is believed that enhancers recruit RNAPII to promoters. Orthogonal evidence that RNAPII is present at high levels at active enhancers and that eRNA expression precedes transcription of protein-coding genes supports this theory (13–16). Dynamic local protein milieus, variable histone modifications, and non-coding elements must all interface in time and space to ensure efficient, inducible transcription. This is enabled by refined chromatin structures.

While it has long been understood that the chromatin architecture is purposeful, the role of this reproducible, predictable structure extends beyond the compaction of DNA. Protein complexes and histone modifications at close-range and various forms of chromatin loops at long-range are all argued to play roles in gene regulation. Having established the core functioning of immune gene regulation, it follows that if chromatin structure is to be shown to influence such regulation it must facilitate robust induction and heterogeneity. Accruing evidence demonstrates that features of chromatin structure at a range of scales are central to effective immune regulation. This includes creating permissive environments for rapid gene induction and modifying chromatin structure to encode epigenetic memory and prompt cell activation. An interface exists between predictable processes and random events which govern chromatin structure creating a potential space for heterogeneity and inducibility. This shows clearly that chromatin structure does parallel the needs of immune regulation, and, therefore, provides another avenue of regulatory leverage which could satisfy the needs of the immune response.

Intrinsic to this tension is the potential for things to go awry. While stochasticity hemmed-in by well-regulated processes creates an environment of diversity and flexibility, hyper-activation or irregular gene expression patterns are a risk. Auto-immunity is a key example of this: misappropriated control mechanisms of the gene regulatory space give rise to seemingly irreversible shifts in expression patterns which are pervasive.

Immune cells are present in virtually all tissues, and both tissue-resident and circulating immune cells play an important role in the immune response. However, these two populations of cells differ in origin, function, and transcriptional program. While circulating macrophages, for example, have a short life-time (up to 1 week) and are continuously replenished from hematopoietic stem cells in bone marrow; microglia - brain-resident macrophages - are derived from the embryonic yolk sac and are replenished locally due to an intrinsic self-renewal capability (17). Due to this, microglia have an extremely long lifespan. It follows that phenomena associated with epigenetic reprogramming, such trained immunity and cell exhaustion, influence the function of these cells for the duration of their lifespan. In general, tissue-resident macrophages display an M2-like phenotype and play an important role in tissue homeostasis (18). For instance; microglia contribute to neuron pruning and neuro-development by expressing the CX3CR1 gene. CX3CR1 knockout mice demonstrate reduced dendritic spine pruning, abnormal synapse maturation, decreased functional connectivity and display behavioral abnormalities (19). As tissue-resident immune cells have unique functions and expression profiles, it is unsurprising that they have unique regulation mechanisms and chromatin structure. PU.1 is a central lineage-determining transcription factor (LDTF) for microglia as well as circulating macrophages. In contrast, IRF8 is a key TF for microglia, but not circulating macrophages (20). In addition to the unique expression of key TFs, nucleosome position and histone modifications in tissue-resident macrophages reflect the unique demands of the surrounding tissue environment. Interestingly, transplantation of macrophages to other tissue sites results in the reprogramming of nucleosome position; suggesting that nucleosome position is determined by local microenvironments likely through cell-cell communication between macrophages and tissue-specific cells (21). Variation in long-range chromatin interactions between tissue-resident and circulating immune cells is largely unknown due to technical limitations of exploring this *in vivo*. Understanding these differences is essential to developmental epigenetics. For example, both microglia and circulating macrophages are considered macrophages, sharing numerous features, although their origin and developmental pathways are different.

In this review, we will discuss the contribution of chromatin structure to immune regulation by exploring how it enables inductive capacity and heterogeneity. Interrogation at single-cell resolution and improvements in chromatin conformation capture techniques posit an array of mechanisms by which chromatin structures regulate immune genes. Through this, we will address two overarching questions: first, which elements of chromatin architecture regulate immune responses and how this is achieved. And, second, how rapid and versatile immune responses facilitated by chromatin structure underlie immune-mediated conditions, like auto-immunity.

### Chromatin Structure as a Property and a Driver of Transcriptional Regulation

Discovery of the staggeringly complex nature of chromatin organization has been fuelled - and limited - by approaches capable of reliably identifying components of 3-dimensional nuclear architecture. Chromatin conformation capture techniques, notably Hi-C and its derivatives, have begun to clarify this area of cellular biology and, in doing so, uncover the role of nuclear organization in controlling gene expression - an area in which there remains active debate. This control takes place at an impressive range of scale and by manifold, seemingly independent, mechanisms. We argue that this regulatory platform created by complex chromatin structures creates a dimension of control which is essential for immune functionality.

#### The Global Organization of Chromatin

**Figure 1:**
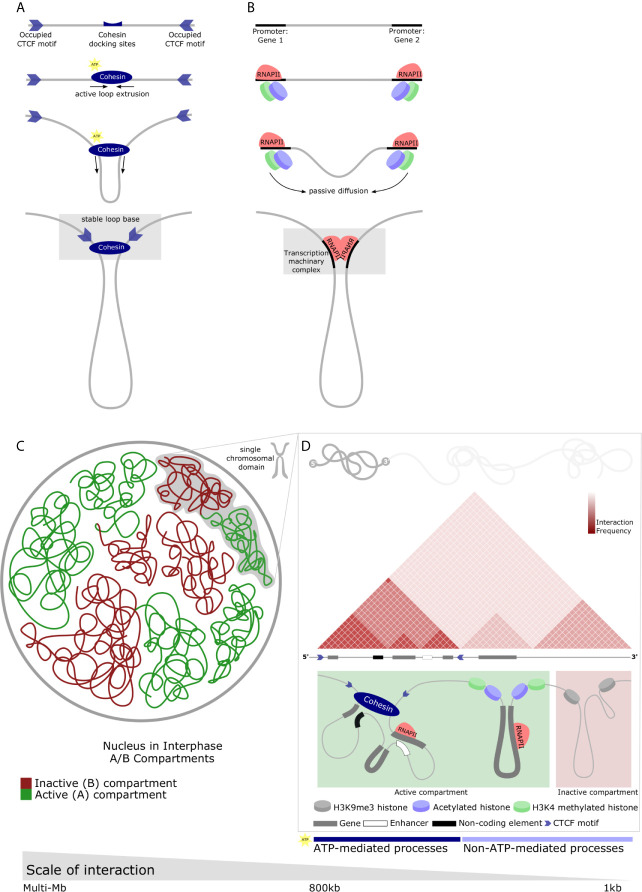
Diverse chromatin structures across the range of nuclear scale and the main proposed regulatory mechanisms which underlie chromatin structure. **(A)** CTCF-mediated looping forms TADs. Directional CTCF motifs across the genome are occupied by CTCF in relation to loading/unloading dynamics of nuclear proteins. Similarly, Cohesin binds chromatin (top panel) and extrudes chromatin through its closed ring structure to form a loop (middle panels). Extrusion continues bidirectionally until convergent CTCF occupied sites are encountered. Heterodimerization of CTCFs, Cohesin, and various other proteins form a stable loop anchor complex (bottom panel) and are the basis of most stable TAD structures. **(B)** RNAPII-mediated looping forms small chromatin loops. TFs bind motifs in the regulatory elements of genes along with RNAPII (top-middle panel). Passive diffusion and random motion which are not ATP-mediated bring these protein structures into proximity with one-another (middle-bottom panel) and, by means of affinity-interactions, chromatin loops form between regulatory elements (bottom panel). These protein-protein interactions may result in liquid-liquid phase separation and the relatively dynamic recruitment of transcriptional machinery, creating a permissive environment for rapid and robust transcription. **(C)** Nuclear compartments are complex chromatin structures. In interphase, the nucleus is divided into A and B compartments. These domains span multiple megabases and comprise active (gene-dense, euchromatin) and inactive (gene-scarce, heterochromatin) possibly mediated by liquid-liquid phase separation. Green - active or A compartment; red - inactive or B compartment. **(D)** Multiple different chromatin loop structures are contained within single chromosomes which contribute to A or B compartments. Individual chromosomes can contain contiguous regions of active or inactive chromatin which represent the interactions of various elements of the genome facilitating functional regulation. These chromatin interactions are depicted by a schematic of a Hi-C map demonstrating a small area of a single chromosome in interphase. Triangular regions of varying red intensity represent the likelihood of interactions between chromatin regions in a population of cells. TAD with boundaries occupied by convergent CTCF motifs insulate areas of the genome (left). Similarly, the active compartment contribution of this representative chromosome may house RNAPII-mediate loops mediating interactions between genes, or genes and non-coding regulatory elements (as described in **B**) (middle). The inactive compartment contribution may be mediated by protein-mediated liquid-liquid phase separation to form repressive compartmental domains in gene-scarce heterochromatin (right). These processes can be either ATP- or non-ATP-mediated.

How it is that 2 meters of DNA is folded within a nucleus less than 20 um in diameter is a long-standing question of human biology. Despite being dynamically altered depending on biological events, such as cell-cycle progression and differentiation, genome structure is not random. Stretches of DNA fold in sophisticated arrangements without entanglement. The nucleosome is the most basic scaffolding agent, as described above, with both structural and functional relevance. At a global level, however, the arrangement of nuclear content is becoming less opaque. The nucleus at interphase is divided into compartments comprising gene-rich active regions and gene-scarce inactive regions, termed A and B compartments respectively (22) (**Figure 1A** **)**. Characterization of A/B compartments has led to the promulgation of liquid-liquid phase separation (LLPS) as the governing force of these large distinct zones which, canonically, represent the largest unit of chromatin association and organization with functional relevance. As technologies have matured, numerous groups have described so-called higher orders of chromatin arrangement. Topologically Associating Domains (TADs) constitute large, looping territories which are stable across cell types (23–25) (**Figure 1B** **)**. The fundamental difference between chromatin compartments and TADs is size; while compartments are multi-megabase in scale, TADs are generally regarded as being between 0.2 and 0.8 megabases in size. In addition to size, chromatin compartments comprise interacting genomic regions on the same or different chromosomes which are in the same transcriptional state - i.e. either expressed or repressed genes. TADs, on the other hand, interact preferentially with themselves and insulate regions of the genome together with (or apart from) neighboring regulatory elements and functionally associated genes (26). An important further distinction is their conservation across cell-type: TAD boundaries are almost always the same [although debate arises here as well; reviewed by Eres and Gilad (27)], whereas A/B compartments are highly variable.

#### Small, Variable Domains of Chromatin Structure Facilitate Heterogeneity and Inducibility

The essential component of heterogeneity for immune function suggests is cell-to-cell variation in gene regulation and, similarly, in chromatin structure. Recent single-cell investigations have shown this heterogeneity unequivocally. The knowledge that chromatin moves dynamically and forms heterogeneous structures is not new. Structural changes of the chromosomes throughout the cell cycle, passive diffusion of chromatin at interphase, and the structural differences of the X chromosome have been explored for decades (28, 29). However, this work has been limited - focusing only on specific loci or operating at poor resolution. Recent single-cell analyses have demonstrated genome-wide heterogeneity with chromatin structures that respond to cell need. Single cell sequencing has convincingly shown that nucleosome positioning is not consistent between cells in a population (30–32). Heterochromatin domains comprising phase-separated droplets are now known to be more dynamic than previously assumed (33, 34). Chromatin interactions are similarly variegated: the frequency of DNA loop formation is approximately 30% of alleles at maximum depending on the locus and RNAPII-mediated loops are heterogeneous (35–37). Likewise, there is diversity between TADs regarded to be conserved across cell-type and species (35, 38–40). Evidently, chromatin structure is dynamic and highly heterogeneous across the range of scale. These findings raise important questions: why is chromatin structure so variable and how is this achieved?

Consistent improvement in technologies which reliably interrogate chromatin interaction networks, the recognition that there appear to be smaller sub-TAD regions within largely static TADs, and the immensity and apparent stochasticity of A/B compartments have all strengthened the case for smaller, more variable components of chromatin structure. These components could reasonably contribute both towards gene expression regulation and DNA compaction. The source of heterogenous genome structures may be derived from the dynamic nature of the genome throughout the cell cycle. Unsurprisingly, distinct cell-cycle stages reveal distinct chromatin structures. However, even within the same cell cycle stage, individual cells harbor some degree of distinct chromatin structure variability. Two interacting components govern this variability. Firstly, ATP-dependent directional events which are unaffected by thermodynamic fluctuations, such as nucleosome depletion by chromatin remodeling complexes and partitioning by actin (41, 42). Secondly, ATP-independent non-directional events which include nucleosome deposition by histone chaperone, the binding of proteins to DNA, and passive diffusion of chromatin (28, 43, 44).

Many long-range DNA loops are protein-mediated with a number of important proteins being implicated in their formation. This mechanism represents a confluence of directional and non-directional events. The most extensively studied DNA-loops are CTCF-mediated loops. Generally housed within TAD boundaries, these loops are appreciated by punctate signals at the summits of associating domains in Hi-C maps representing the close association of structural proteins (26, 45). The loop extrusion model is posited as the dominant theory of how these domains are formed describing their active formation by SMC proteins, especially cohesin complexes (**Figure 1A**) (46–49). In brief, these proteins dock and travel divergently along sequences of DNA, extruding chromatin through their looped structure, until they reach TAD boundary - occupied CTCF sites which are orientated in the correct manner. Thereafter, a stable loop ‘base’ is formed through, amongst other processes, the heterodimerization of CTCF and Cohesin rings which secures the TAD. This is supported by convergent CTCF-binding sites being a prominent feature of TAD boundaries and it is now largely agreed that convergent CTCF-binding motifs are the predominant boundary element required for TAD formation (48, 49). This mechanism explains the non-overlapping property of TADs and the predictable location of aberrant TADs following CTCF deletion (45, 48).

There remains debate as to the intricacies of this mechanism and it is incompletely understood. For example, CTCF sites arranged in tandem likely form loops of a differing structure which could contribute to the topology of a domain. Additionally, the distribution of genes within CTCF-loops is seemingly determined by their being either constitutive or cell-type specific with the implication that genes localized within the loop must have a means of induction when needed (50). There also appears to be a distinct role for Cohesin complexes and CTCF; perturbation of the functional pathway of either results in different, and sometimes contradictory, effects on chromatin structure (26). However, depleting CTCF or attenuating Cohesin loading result in the near-complete loss of TADs and CTCF-loops (51, 52). Surprisingly, this enhances compartment strength without having a major effect on baseline gene expression (52). Nor does cohesin depletion affect histone marks while a small number of active genes are repressed (53). Thus, an important discovery is that the action of Cohesin, and therefore the role of CTCF-loops, may be implicated in responses to stimuli and lineage commitment as opposed to gene expression at steady state. This is plausible as a number of enhancers and the genes they induce are co-localized by CTCF-looping. Indeed, Cohesin was shown to be necessary for the induction of responsive genes and enhancers in myeloid cells and macrophages exposed to inflammatory stimuli (54).

RNAPII is another example of protein-mediated DNA looping, specifically between promoters and enhancers (55). Unlike CTCF-loops extruded by ATP-dependent means, RNAPII facilitates interactions by affinity and, therefore, does not irreversible characteristic of CTCF-loops. Instead, RNAPII-mediated loops are driven by cell activity. RNAPII, by facilitating interactions between genes and enhancers within a CTCF-loop and an active enhancer or promoter at a loop anchor, may drive the expression of tissue-specific inducible genes in a coordinated manner with house-keeping genes (**Figure 1B**) (50). Proteins within the nucleus, therefore, have an established role in the formation of chromatin structure. The interplay of ATP-dependent mechanisms (CTCF-loops) and affinity-driven mechanisms governed by protein kinetics (RNAPII-loops) creates potential for inducible gene expression and rapid responses. Furthermore, proteins create a distinction in location between active cell-type specific genes, constitutively active non-specific genes, and active enhancers suggesting that chromatin structures at this level may enable versatile responses.

Heterogeneity thus relates to processes of chromatin formation and movement. However, stochasticity is inevitable and influences even relatively stable directional processes. For example; the loop extrusion process by SMC proteins itself is not influenced by thermodynamic instability, however, the timing and start location may be stochastic owing to cohesin docking. The end location is, likewise, stochastic: cohesin continues extruding DNA until blocked by CTCF and released by cohesin-releasing factors. TFs and CTCF - which hamper cohesin’s advancement - repeatedly bind and unbind DNA with CTCF remaining mostly in the unbound state (56, 57). Therefore, cohesin may advance past CTCF-binding motifs resulting in heterogeneous loop lengths and variable boundaries. Indeed, the average size of CTCF-loops increases following CTCF deletion (58–60).

There are further components to the non-directional forces driving heterogeneity within chromatin structure. Just as TADs comprise a combination of small protein-mediated loops which scaffold the nucleus through directional events, so A/B compartments are likewise a composite of smaller domains (**Figure 1C**). However, the formation of these DNA loops is stochastic. Compartmental domains of self-associating chromatin form by non-directional forces, frequently containing a few (or even a single) gene loci(us) and the associated regulatory sequences (61). The most frequent interactions are those between transcriptional start- and end-sites of transcribed genes (61). This drives an accumulation of transcriptional machinery and TFs and there appears to be a clear link between transcription and compartmental domain formation. In-line with this, inter-compartmental domain associations appear stochastic and their strength and frequency reliant on the concentration and type of associated proteins in the looped region (33). Therefore, the affinity and the interaction of these protein complexes will determine the cooperativity of chromatin regions. Compartmental domains comprise either active or inactive chromatin replete with differing sets of multivalent protein complexes could interact preferentially with one another in a manner representative of A/B chromatin compartments (26). Indeed, phase separation owing to multivalent TF cooperativity could occur (62, 63). Supporting this model, phase separated droplets of chromatin may be formed by protein complexes with intrinsically disordered domains including RNAPII and TFs (64, 65). Notably; hyper-phosphorylated - active RNAPII - has been shown to form droplets (66). It would follow that this process would form domains of active chromatin. On the other hand, heterochromatin is occupied by a starkly different complement of histone modifications. For example, H3K9me3 is recognized by the protein HP1, recruiting the specific histone methyltransferase for H3K9 (SUV39H1) *via* the DNA methyltransferase DNMT3A and the DNA methylation binding protein MECP2 (67). As SUV39H, in turn, transfers methylation group to its neighboring histones H3K9me3 marks are propagated and form tightly packed, functionally silenced heterochromatin which is likely to associate with other such regions (**Figure 1D**). While CTCF-loops adhere to TAD boundaries, compartmental domains appear to be less restrained. CTCF-loops appear to insulate compartmental domains of the same or different compartments therefore modulating interactions among compartmental domains at close range by a directional force (26). Long-range contacts between compartmental domains, however, are under the influence of thermodynamic principles.

A balance thus exists between two independent organizational events which suggests a complex and reciprocal relationship between transcription and genome architecture (52, 68). The non-directional formation of compartments adds a degree of complexity when in concert with ATP-dependent, protein-mediated mechanisms. The accumulation of proteins required for transcription in space and time creates a permissive environment for rapid and robust transcription. Additionally, the formation of phase separated structures requires constant fusion and fission of droplets creating a dynamic chromatin structure capable of versatility.

### Inducible Immune Responses: Rapid and Refined Gene Expression Encoded by Genome Structure

#### Rapid Response Genes Are Prepared for Robust Action by Nucleosome Modification in Innate Immune Cells

A functioning repertoire of innate immune cells requires that they are rapidly and efficiently activated to enact effector functions, such as responding to invading pathogens. This is reliant on a change in epigenome to facilitate a transcriptional shift. Nucleosome remodeling functions as a gatekeeper in this process: determining which enhancers and promoters become nucleosome-free and regulate immune gene transcription. Nucleosome position is determined both by cellular signaling and developmental programming (69, 70). Stimulation by external signals induces TF cascades resulting in remodeling; these are termed signal-dependent transcription factors; SDTFs. On the other hand, developmental regulation of nucleosome position is informed by LTDFs (71, 72). PU.1 (SPI1) is a known LDTF in both innate and adaptive immune cells - T cells and macrophages (73). PU.1 is also a pioneer factor able to bind DNA in spite of nucleosome presence at binding sites and recruits chromatin remodeling complexes (74). However, PU.1 is not sufficient to induce transcription, rather, it binds cooperatively with other TFs such as AP-1 and members of the IRF family, which are essential in inducing transcription (75, 76). In this way, PU.1 functions as a safeguard to prevent unwanted transcription in the absence of other essential TFs (77).

To induce signal-dependent immune genes, SDTFs, such as NFkB, play a key role. NFkB is expressed constitutively, forming a complex with the repressor protein IκB and is present in the cytosol in this form (78). Both immune and barrier cells, such as epithelia, recognize pathogens through cell-surface receptors such as Toll-like Receptors (TLRs) and C-type Lectins. This recognition triggers the phosphorylation and subsequent degradation of IκB, prompting NFkB nuclear translocation after which it occupies proximal promoters on immune genes. In turn, transcription is induced by the recruitment of co-activators and P-TEFb (6, 79).

Transcription of rapid response immune genes is detectable within 15 minutes following pathogen recognition (80). Despite the first step in transcription induction for most immune genes being nucleosome depletion at promoters, the promoters of numerous immune genes are depleted in resting, or naïve, states. Indeed, it has been shown that knockdown of the SWI/SNF complex does not negatively influence rapid response gene expression, despite other genes being suppressed (81). Even more striking is that not only is nucleosome depletion a feature of naïve immune gene promoters; pre-loading of RNAPII has also been observed at these sites (82). This is an important observation as nucleosome depletion may take minutes (83). Indeed, it has been shown to take ~30 min to achieve nucleosome depletion at immune genes promoters (84). Some active histone marks such as H3K4me3 are also found at rapid response gene promoters at naïve state (85). Elongation marks such as histone acetylation and P-TEFb are not found at naïve state, but P-TEFb is in turn recruited by the bromodomain-containing protein Brd4, which detects H4K5/8/12Ac inducibly acquired at rapid response gene promoters (86). Given that first response genes become detectable within 15 minutes, the benefits of removing the nucleosome prior to immune challenge is apparent. This is demonstrated in Innate Lymphoid Cell (ILCs) (87). ILCs, including NK cells, which develop from lymphoid progenitors, express cytokine genes similar to innate immune cells. Of the 3 subtypes of ILCs, nucleosomes at regulatory elements of IFNg are only depleted in group 1 cells. This depletion is regulated developmentally and removed in a stepwise manner. In addition to nucleosome depletion, p300 is present at rapid response gene promoters in the naïve state, however, p300 shows stronger binding after stimulation. Although nucleosome depletion at rapid response genes is developmentally regulated, it remains unknown how nucleosomes are depleted from rapid response genes only. Rapid response gene enhancers are identified by H3K4me1 enrichment in the naïve state (88). To prevent transcription in this state, repressive histone marks, such as H3K9me3, H3K27me, and H4K20me3 are deposited at the enhancer together with the co-repressor recruitment complex Bcl6 (89, 90). In another study, transcriptional responses to TLR4 ligation primarily relied on pre-existing H3K4me2 enriched enhancers while *de novo* enhancers are regulated in a PU.1- and NFkB-dependent manner (91).

Contrary to previous dogma suggesting that innate immune cells lack memory capability, histone modifications, such as the addition of H3K4me3 at gene promoters, have been shown to infer long-term memory to past infectious challenges (92). This phenomenon, referred to as trained immunity, increases the speed and efficiency of rapid response gene transcription. The most prominent example being circulating macrophages. Following detection of BCG or beta-glucan exposure through C-type Lectin Dec1, their transcriptional profiles are durably altered; enabling robust responses to future immune challenge. Simultaneously, their metabolic profiles are altered. Upregulation of mTOR/HIF1a by Dec1 signaling activates glycolysis, glutaminolysis, and cholesterol synthesis pathways while oxygen consumption is reduced (93). This shift towards an aerobic glycolytic predominance is observed even under high-oxygen conditions. The role of this is the synthesis of intermediate metabolites, rather than maximizing energy production. Fumarate, an intermediate metabolite of the TCA cycle, is known to inhibit the histone demethylation activity of KDM5 (94). Due to the higher concentration of fumarate following mTOR/HIF1a upregulation, demethylation of H3K4me3 at some inflammatory gene promoters, such as IL6 and TNFa, is inhibited. This maintains H3K4me3 at these promoters even after stimuli are removed. Additionally, alpha-ketoglutarate is a cofactor of KDM5 and thus the balance between fumarate and alpha-Ketoglutaric acid influences KDM5 activity (94). Likewise, glutamine synthesized by glutaminolysis is converted to citric acid and its influx into the TCA cycle contributes to the accumulation of fumarate. Cholesterol synthesis is another pathway implicated in enhancing trained immunity; mevalonate, a cholesterol intermediate, contributes to the accumulation of H3K27ac; the precise mechanism of how this occurs remains unclear (95).

Strikingly, this epigenetic reprogramming can occur at the hematopoietic stem cell level, giving rise to trained macrophages which persist for at least 3 months (and perhaps even decades) (96, 97). Conversely, the addition of repressive chromatin marks leads to a slower, attenuated immune response. Chronically activated immune cells lose their ability to produce cytokines, proliferate, and lyse pathogens. The inflammatory gene promoters in these cells are not acetylated by the 2nd challenge (98). These phenotypic features revert to the naïve state following exposure to beta-glucan (99). Therefore, the speed and success of the immune response is fine-tuned by the dynamic modification of histone marks.

#### Enhancer-Promoter Interactions Are Pre-Formed at Rapid Response Genes in The Steady State in Both Innate Cells and T Cells

Successful rapid responses are enabled by long-range genomic interaction as well as local structures, such as nucleosomes. Enhancers-promoter loops at rapid response genes are pre-established in the naïve state (100). These enhancer-promoter interactions are mediated by both sequence-specific binding proteins, such as CTCF, and non-sequence-specific proteins, such as RNAPII. However, it remains unknown how the loops themselves are established. Interestingly, while immune cell activation leads to nucleosome remodeling at non-rapid response genes, most of the enhancer-promoter interactions at these genes are pre-established. Promoter capture Hi-C (PCHi-C) experiments have shown that activated macrophages (both M1 and M2 polarized) display very similar enhancer-promoter interaction patterns to naïve macrophages (M0 macrophages) (101). Despite this apparent stability of long-range interactions, enhancer-promoter loops are known to be changed by activation. In THP-1 cells, for example, gained enhancer-promoter interactions have been identified by PCHi-C (102). The reasons for the discrepancy in findings between studies which suggest long-term stability being a feature of enhancer-promoter loops and those providing evidence of dynamic change associated with the functional state of cells is still a topic of debate. One possible explanation relates to the dynamics of the genomic structure. PCHi-C experiments in macrophages were performed at 24 hours after activation as opposed to at 4 hours in THP-1 cells. If observed structural features of the genome are emergent properties of a self-organizing system, genome structure would be perturbed temporally by stimulation before returning to stable steady state within 24 hours (103). Despite difficulties in ascribing strict rules to enhancer-promoter interactions, it is clear that these patterns are highly cell-type specific and immune activation minimally alters these interactions, at least in the case of rapid response genes. Therefore, the strategy of pre-forming loops during steady or naïve states to prepare for transcriptional responses is not immune cell-specific. Rather, this is the general strategy employed by responsive genes and occurs in a signal-dependent manner. For example, glucocorticoid receptor response genes also form enhancer-promoter interactions at steady state (104).

Correlations between loop formation and cellular activity states does not suggest that long-range interactions regulate the timing of responsive expression. However, forming a loop before activation seems biologically reasonable. Cohesin needs nearly 40 minutes to form 900 Kb loops by extrusion (53). Considering that the average TAD is 1 Mb, reconstruction of the chromatin interaction pattern requires at least 40 minutes. Furthermore, various DNA-binding proteins present in the nucleus form large complexes before being functional. When cohesin encounters these DNA-binding proteins, loop extrusion takes place at a slower rate than naked DNA *in vitro* (105). If chromatin re-organization facilitates relatively slow cellular responses, such as cell-cycle progression or differentiation, this is acceptable. However, if rapidity is essential to the response in question, as in immune responses, it would be detrimental to dramatically re-wire enhancer-promoter interactions at every challenge to allow for rapid gene responses. Further work is needed to decipher the relationship between enhancer-promoter interactions and the control of expression timing. An approach might be to investigate the influence of depleting chromatin re-organizing factors on the timing of gene expression. For example; Batf is a pioneer factor which recruits CTCF *via* the Ets protein family (106). If enhancer-promoter interactions do control expression timing, the deletion of Batf would delay the onset of transcription at affected sites.

**Figure 2:**
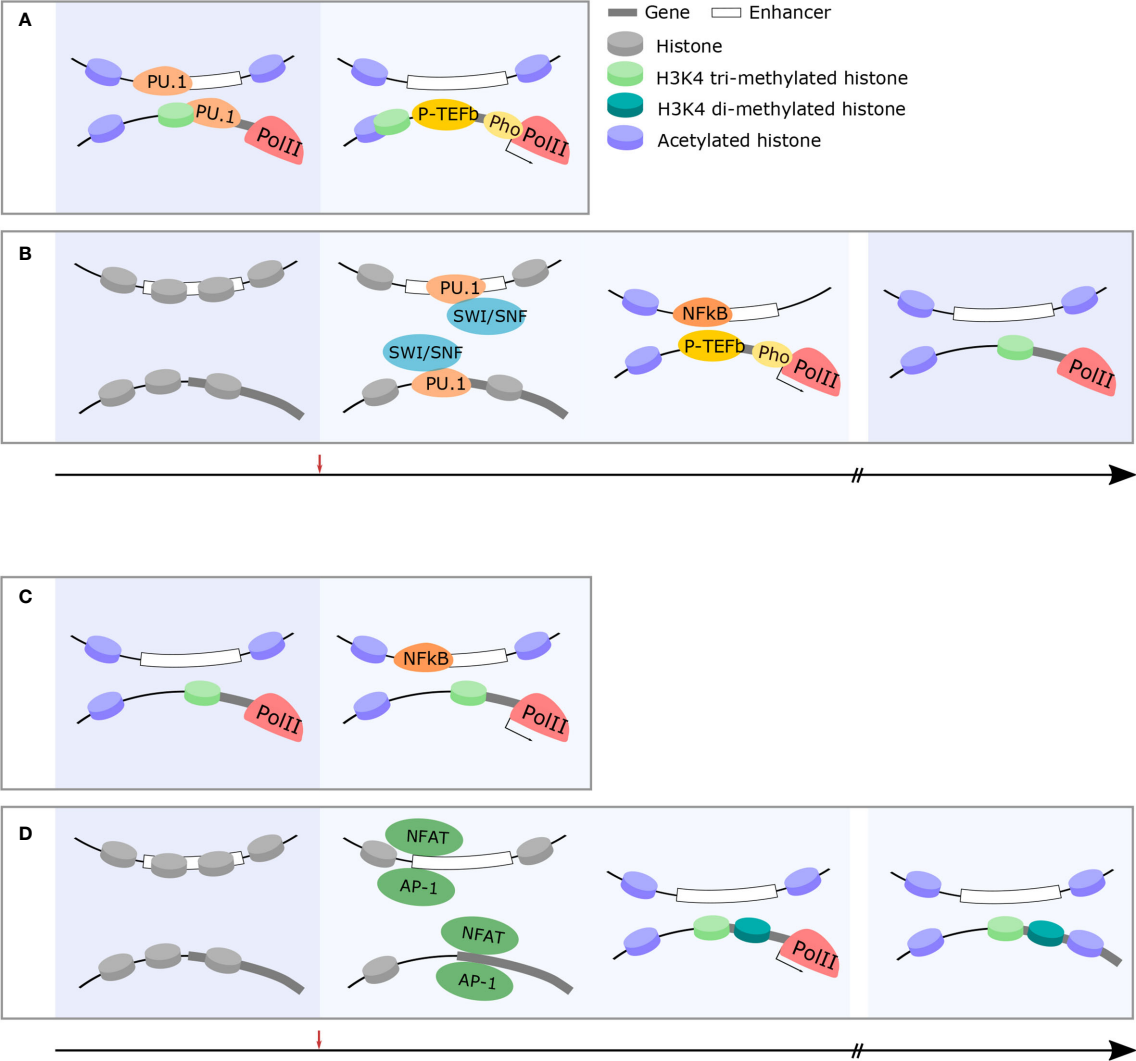
Immune cells exhibit changes in chromatin structure which facilitate rapid, robust responses and facilitate long-term memory. **(A)** Rapid-response genes in innate immune cells are primed for rapid and robust responses in the naïve state. Permissive chromatin marks, acetylation and H3K4 trimethylation, at gene promoters, facilitate the pre-loading and stabilization of RNAPII at rapid-response gene promoters (left panel). Following challenge, P-TEFb phosphorylates RNAPII and active transcription takes place within minutes (right panel). **(B)** Early-response immune genes are primed following immune challenge and maintain epigenetic memory to facilitate rapid and robust responses in future. In the naïve state, early-response immune gene loci are inaccessible chromatin structure (left panel). Following immune challenge, pioneer TFs (PU.1) recruit the SWI/SNF complex (middle-left panel) which, in turn, histone-modifying enzymes and facilitates the recruitment of permissive histone marks and immune-specific TFs (NFkB) initiate the transcription cascade (middle-right panel). This occurs within 30 min and the epigenetic modification of these loci is maintained to facilitate rapid and robust responses in future (right panel). **(C)** Rapid-response genes in T cells are primed for rapid and robust responses in the naïve state. Similarly to innate immune cells, permissive chromatin marks, acetylation and H3K4 trimethylation, at gene promoters, facilitate the pre-loading and stabilization of RNAPII at rapid-response gene promoters (left panel). This allows for rapid transcription of these genes within minutes following immune challenge even in adaptive cohorts of cells (right panel). **(D)** T cell effector genes are epigenetically modified over time following immune challenge to facilitate long-term immune memory and more effective responses following future challenge. In the naïve state, effector gene loci are silenced by repressive chromatin marks (left panel). Following immune challenge, the recruitment of immune-specific TFs (NFAT) (middle-left panel) results in histone modification and the recruitment of RNAPII and subsequent expression of effector genes (middle-right panel). In memory states, these specific histone modifications are maintained (right panel).

In summary, rapid response genes are prepared to initiate cellular function in a developmentally-programmed manner by removing nucleosomes, modifying histone tails, and forming enhancer-promoter loops (**Figures 2A, B**). Additionally, constitutive expression of NFkB in the naïve state appears to be an important factor in accelerating transcriptional initiation as it omits the need to rapidly transcribe the NFkB gene. However, there remain unanswered questions. LDTFs such PU.1 play an important role in depleting nucleosomes during developmental programming and yet not all PU.1 binding sites situate at rapid response genes. It is likely that other TFs which cooperatively bind PU.1, such as AP-1 and the IRF family, are important in selecting rapid response genes and, thus, control the timing of transcription. Likewise, it is unknown how sites for pre-formed loop formation are selected. Finally, not all inflammatory genes are trained by beta-glucan or BCG treatment and it remains unclear how specific genes are selected. Previously our group identified a novel class of lncRNAs called Immune Priming LncRNAs (IPLs). We showed that UMLILO, an IPL located upstream of IL8, is essential for training the IL8 gene (107). Non-coding transcripts, such as eRNAs, likely play an essential role in defining trainable genes.

#### Genomic Structure and Immune Activation in T Cells

Macrophages and T cells differ markedly in the activation pathways they follow. In the case of macrophages, interaction between pathogen components and pattern recognition receptors is sufficient to initiate effector responses, such as cytokine production. On the other hand, T cells require multiple steps for successful activation. Firstly, pathogen recognition by T cells relies on antigen presentation by antigen presenting cells - such as dendritic cells - followed by secondary stimulation. For example, CD4+ helper T cells use CD28 to recognize CD80 and CD86 on dendritic cells. Once activated, T cells enter a proliferative state undergoing clonal expansion and differentiate into various effector cell repertoires depending on the local cytokine milieu. Th1 and Th2 are the predominant subtypes of activated CD4+ helper T cells and play important roles in host defense and humoral immunity, similar to M1 and M2 polarized macrophages, respectively. In the case of CD8+ cytotoxic T cells, the majority of activated CD8+ cells become short-lived effector cells. A subset of CD4+ and CD8+ T cells become long-lived memory T cells enabling a rapid and specific adaptive response on subsequent challenge. The mechanism of memory T cell development remains controversial (108). Owing to the multiple steps involved in activation, proliferation, and differentiation, a fully-fledged T cell response occurs in the order of days; unlike macrophages which are activated within hours. It follows that T cells have sufficient time and capacity (through their sophisticated differentiation programmes) to restructure their genome, as opposed to innate immune cells. Here, we discuss the alterations in genome structure occurring in T cells following immune challenge. Changes to genome architecture at sites encoding the variable T Cell Receptor (TCR) to allow for rearrangement are well-described and reviewed elsewhere. As such we will focus on non-TCR regions with a specific emphasis on cytokine genes.

##### T Cell Activation and Changes in Local Genome Structure

Numerous studies have shown chromatin accessibility to be extensively altered following T cell activation. One of the earliest investigations demonstrates this in the promoter region of Il2 - a growth factor influencing T cell proliferation, survival, and differentiation expressed in both CD4+ and CD8+ T cells at various times post-challenge (109). The Il2 promoter and neighboring enhancers are inaccessible in the naïve, pre-challenge state but are remodeled by SWI/SNF chromatin remodeling complex recruited by NFAT and AP1 after activation (110–113). The role of neighboring enhancers is highly cell type-specific with different enhancers being activated to induce il2 expression in CD4+ and CD8+ T cells (114). Despite this, genome-wide investigation showed that most chromatin accessibility gains are shared between activated CD4+ and CD8+ T cells despite their different effector functions (115). Notably, most chromatin remodeling is complete within 24h as differential peaks at 24h and 72h after activation as probed by ATAC-Seq are largely overlapping (115, 116).

However, in a similar manner to early response genes in innate immune cells, some genes are prepared for activation in the naïve state. Nucleosome depletion and RNAP2 loading along with some active histone marks can be observed at the Tnfa promoter in CD8+ T cells. In contrast, this dynamic change occurs only after activation at the Ifng promoter (117, 118). Interestingly, other early response genes such as Ccl3 and Il2ra are enriched with H3K4me2, but not H3K4me3 as might be expected (117). It remains unknown why not all early response gene promoters are enriched with H3K4me3. It is possible that this is essential for the precise regulation of transcriptional timing.

A subset of CD4+ and CD8+ T cells exhibit transcriptional memory to previous pathogenic encounters which engender rapid and robust transcriptional responses on re-challenge. These memory T cells do so not only by rearrangement of the genomic TCR region, but also epigenetic change at cytokine gene loci, similar to immune training in the case of innate immune cells. Additionally, enhancer regions of the memory-associated genes DUSP10, BCL6, and TNFSF10 become accessible after activation in T cells and these structural changes are stably maintained even after the resolution of an immune challenge (119). Likewise, memory T cell enhancers and promoters are in a poised state demonstrated by the accumulation of active histone marks (120). The mechanism of epigenetic change following activation in this cohort of cells remains unclear. It is possible that, similar to that which occurs in trained immunity, intracellular metabolites in activated CD8+ T cells increase flux through key pathways such as glycolysis and glutaminolysis (121). On the hand, chronic infection is known to lead to T cell exhaustion and this epigenetic landscape is distinct from that of memory T cells (122).

There is ample evidence demonstrating that the dynamic remodeling of chromatin structures and histone modifications are an important characteristic of T cell functionality. A wide range of genes necessary for activated T cells undergo such changes, notably exclusions are those implicated in rapid response such as Tnfa. As in the case of trained immunity in the context of the innate immune response, memory T cells prepare for future responses by remodeling chromatin at cytokine genes (**Figures 2C, D**).

##### T Cell Activation and Enhancer-Promoter Interactions

Some of the earliest and most seminal work on T cell genome structure details the interaction of Ifng and Il4, cytokine genes characteristic of Th1 and Th2 CD4+ subsets respectively (123–125). Ifng and Il4 loci interact in the nuclei of mouse naïve T cells despite being located on different chromosomes. Following T cell polarization, however, this interaction is lost and Ifng, for one, interacts preferentially with enhancers in Th1 cells (126). The interactions of Ifng and enhancer, at least some of them, are mediated by T-bet (127). Furthermore, genome-wide analysis of the interactome at Ifng and Il4 promoters revealed induced interactions with other Th1 and Th2 specific genes (128). Although the majority of the interactions are stable during activation, *de novo* interactions mediated by STAT proteins exist between key genes of the CD4+ response (128).

Further examples of interactions taking place in the genome of T cells are those between Il4, Il5 and Il13 gene loci. These cytokines are transcribed in Th2 cells but not naïve or Th1 cells. Under most circumstances and in many cell types these loci are in contact, fibroblasts being a proven example of this. Interactions with the locus control region (LCR) are also observed in T cells (129). Surprisingly, LCR-promoter interactions occur — although to a weaker degree — in naïve T cells and Th1 cells. This suggests that Th2-associated cytokine genes interact with enhancers to facilitate rapid induction in Th2 cells and thus the expression of Th2-associated cytokines are regulated by the subtype-specific activation of enhancers rather than the interaction (129–131).

Recent genome-wide chromatin interaction studies have identified interactions specific to T cell activation. Interestingly, and similar to their chromatin accessibility profiles, activated CD4+ and CD8+ T cells share the same chromatin interactions despite differences in effector function (115).

Cis-regulatory element interaction studies have intriguing results. One such PCHi-C study showed activated and naïve T cells with similar patterns of enhancer-promoter relationships.

On the other hand, H3K27ac HiChIP experiments have demonstrated that naïve T cells show distinct enhancer-promoter interactions compared to Th17 and regulatory T cells (132). One possible explanation for these discrepancies is methodological disparity. PCHi-C captures all interactions with promoters within the nucleus, including the loops which form to facilitate nuclear compaction, while HiChIP captures primarily active regions identified by H3K27ac pull-down. Therefore, interactions mediated by H3K27ac are underrepresented in PCHi-C data. A further explanation is cell type variety. The biology of T cells is complex; it is plausible that while activated Th1 and Th2 populations have similar genome interaction patterns to naïve T cells, Th17 and Treg cells may show entirely different functional patterns. Furthermore, owing to the fact that Th17 and Treg cells are relatively rare, their interaction patterns may have been diluted by a pooled PCHi-C approach as opposed to cell-sorting occurring in HiChIP approaches.

### Heterogeneity in the Immune Response: Dynamic and Heterogeneous Genome Structures Influence the Transcription and Phenotype of Immune Cells

Expression patterns in immune response are known to be highly heterogeneous. Even in consistent genetic and environmental conditions - for example LPS stimulations in cultured cell lines - phenotype and transcription patterns are distinct at the single cell level following activation (133). *In vivo*, epigenetic modifications by past infection and pathogen variability add further layers of complexity to this heterogeneous response (134). The source of heterogeneity in transcription can be classified as extrinsic or intrinsic noise.

#### The Contributions of Genomic Structure in Robust Gene Expression

Intrinsic noise arises from the random nature of biochemical reactions in the processes of transcription and translation. Transcription is characterized by discontinuous bursts; the interval between bursts produces heterogeneity and cell-cell variability in transcription (135). To limit perturbation by intrinsic noise, cells insulate expression fluctuations by gene regulatory networks (136). For example, the expression of NFkB - a key TF in immune responses - is heterogeneous at steady-state (137). However, absolute expression levels of NFkB at steady state are not a determinant of activation following stimulation; even cells with a dearth of NFkB are activated efficiently. A feedback loop consisting of NFkB and P50 detects fold changes in NFkB concentration in the nucleus, inducing activation rather than absolute NFkB concentrations (137, 138).

There is relatively little cell-cell variation in the expression of genes involved in transcription, such as kinases and TFs, however, cytokine and chemokine expression varies markedly between cells (139). Some cytokine and chemokines are expressed binomially while others are expressed ubiquitously (140). Given the stochastic forces governing chromatin structure and transcription, it is not surprising that the expression of cytokines and chemokines following activation is highly variable. Rather, the intriguing question is how ubiquitously expressed genes, such as IL8, are stably transcribed from an heterogenous chromatin structure. Despite the frequency of enhancer-promoter interaction at maximum approximately 30%, IL8 is expressed in over 80% of innate immune cells following LPS stimulation (35, 133). One explanation is that direct interaction between the enhancer and promoter, which cross-linking based techniques such as ChIA-Drop and Hi-C, detect, is not essential. The majority of TFs have intrinsically disordered regions, enabling the formation of biological condensates (62). Condensates, namely LLPSs, are membrane-less organelles and allow rapid, reversible condensation of specific proteins and nucleic acid into discrete assemblies that dynamically exchange biomolecules with the surrounding environment. Therefore, enhancers and promoters located in the same condensate could interact and induce transcription without direct contact.

Another possible explanation is the presence of multiple enhancers. Frequently, a single gene interacts with multiple enhancers. This is particularly true of robustly expressing genes determining cell identity which interact with multiple enhancers and form so-called super-enhancers (141, 142). In this scenario, only a portion of available enhancers need interact with a promoter and induce transcription. For example, if 10 enhancers can interact with the same target gene at a probability of 30%, approximately 98% of cells will display an interaction with at least one enhancer. In immune responses, NFkB forms such a super-enhancer by exploiting pre-formed chromatin structures; multiple loops are thus important in robustly expressing NFkB (143, 144). Indeed, redundancy of enhancers is a pervasive feature of the mammalian genome and is essential for robustness of expression (145). Indeed, IL8 forms a super-enhancer in macrophages. Importantly, these two features are not mutually exclusive. The LLPS model suggests that condensates are formed at super-enhancers. Therefore, if at least one enhancer is located within a condensate at close range to the gene of interest gene induction can occur. In this situation, stable ATP-dependent loops formed by cohesin and CTCF could be key factors as deletion of CTCF binding sites increases cell-to-cell variation of gene expression (146).

A third possibility for this robustness of immune gene expression can be explained through the lens of dynamics. For example, transcription is known to confine chromatin movement (147, 148). If immune genes are transcribed, movement of chromatin is confined and this facilitates robust transcription. On the other hand, if no transcription takes place, promoters and enhancers diffuse randomly, increasingly their probability of interaction. Therefore, although there is delay, many cells could express immune genes.

Small-scale genome structures such as nucleosome position and histone modification have also found to be associated to heterogeneity in gene expression. Nucleosome position at promoter is varied, especially lowly expressed genes while it is less heterogenous in actively transcribed gene promoter (149). This robustness of nucleosome position is probably due to the stable nucleosome depletion by ATP-dependent chromatin remodeling complex. For histone modification, H3K4me3 signals positively correlate with high gene expression in immune cells and contribute heterogeneity in gene expression (150). It remains unknown how frequently cells (or allele) pose specific histone modification at regulatory elements of rapid response gene precisely and how histone modifications contribute robustness and heterogeneity in gene expression. Ideally, multi-omics data which detects gene expression and histone modification simultaneously should be applied to elucidate it. Although these techniques are recently developed, the inference of data is not straight-forward to fully understand relationship between histone modification and gene expression (151). A lot of histone modification have been identified and studied relationship with gene expression and they are inter-related mutually. For example, H3K9ac and H3K27ac are associated with active gene expression, but it remains unknown whether H3K9ac is always co-localized with H3K27ac, and whether H3K27ac only promoter shows distinct robustness in gene expression from H3K9ac/H3K27ac co-localized promoter. To understand contribution of histone modifications in gene expression robustness, it must consider gene expression probabilities under specific condition represented by histone modification pattern. Therefore, Baysian approach will be useful for inference of data.

#### The Influence of Genomic Structure on Cell Fate Following Activation

**Figure 3:**
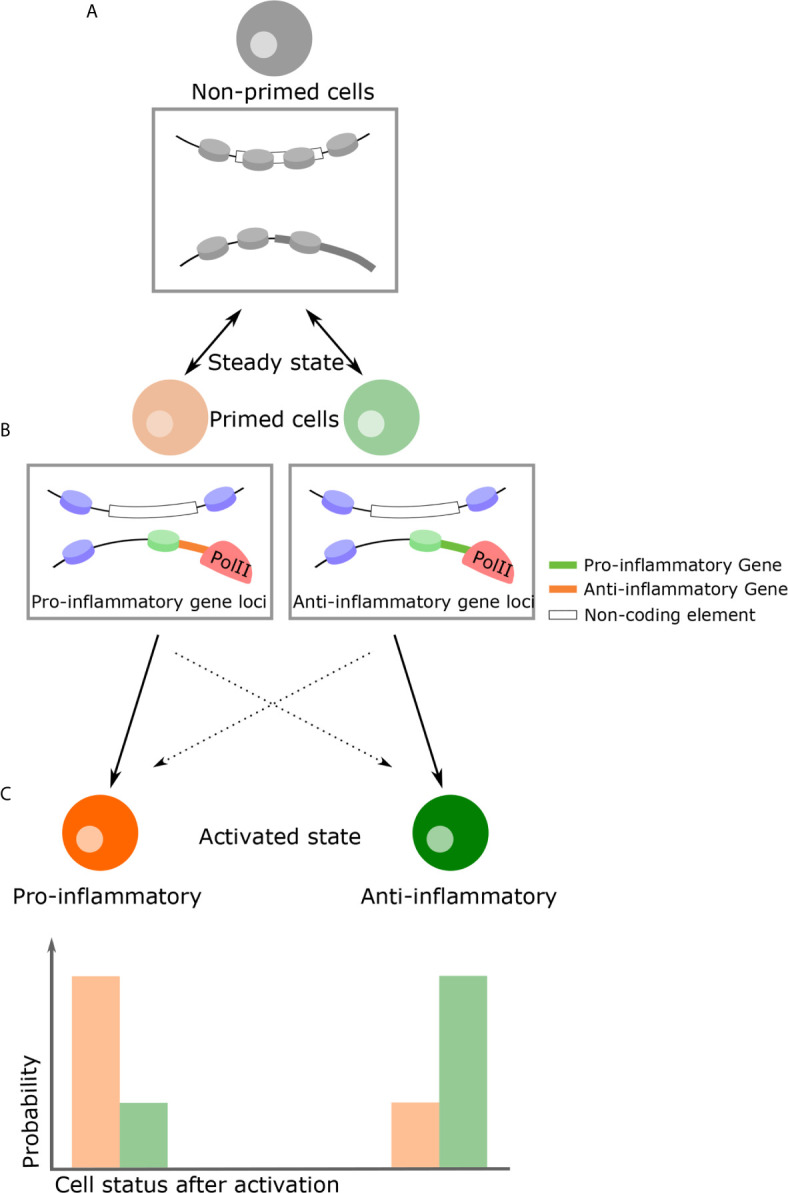
A proposed model of how genome structure influences phenotypic consequence. **(A)** In the naïve state, all inflammatory cells are almost indistinguishable with regards to their transcriptional profiles and durable epigenetic changes have yet to take place. **(B)** Due to thermodynamic fluctuations, however, some cells become primed states transiently at various possible loci. Chromatin structural changes by means of external signals, for example NO as a paracrine signal, can favor a primed state with specific characteristics. **(C)** If cells encounter activation signals in this primed state, there is a high probability they will become a specific phenotype following activation. For example, pro-inflammatory primed cells become pro-inflammatory cells (and vice versa). On the other hand, owing to the intrinsic stochasticity of all transcription, primed cells can become the opposing phenotype (i.e. inflammatory or anti-inflammatory), although this probability is lower. Rectangular insets below cells diagrams is a representation of the chromatin changes which might occur at specific gene loci.

In the steady-state, populations of THP-1 are not transcriptionally distinct. However, following LPS challenge, M1 and M2-like phenotypes develop despite variation in expression levels due to intrinsic noise (133). If phenotypic decision after stimulation is explained solely by cell-cell variation in gene expression arise from intrinsic noise, the ratio of the M1 and M2-like phenotypes are always constant. However, in macrophages, nitric oxide (NO) adjust and limit overall inflammation intensity, influencing decisions on phenotype (152, 153), suggesting that extrinsic noise such as paracrine signaling influence on phenotypic decision making. Analogous to quorum sensing observed in bacteria, cells detect and respond to population density through cell-cell communication. For example, T cells use IL2 as an autoinducer that regulates population density through STAT5 signaling (154–156). In plants, NO affects chromatin structure, suggesting that alteration at steady-state by NO influences phenotypic decisions (157). Likewise, in many biological processes, such as in differentiation, alteration of chromatin structures precedes expression reprogramming (158–160). This implies that even in cells with the same transcriptional pattern, cell fate could be determined by differing chromatin structures. Therefore, it is possible that heterogeneous chromatin structures at steady-state influence phenotype, despite being apparently indistinguishable in gene expression. Of note, chromatin structure is a probabilistic modulator of gene expression as opposed to an absolute determinant. For example, enhancer-promoter interactions regulate the frequency of transcription bursts but do not eliminate stochasticity (161). This principle also applies to the nucleosome and not all genes with nucleosome-free promoter are transcribed (30). Therefore, specific chromatin conformations at steady-state may increase the probability of M1 (or M2) phenotypes following stimulation, but this is not deterministic (**Figure 3**).

Due to technical limitations in study, the influence of immune cell heterogeneity in phenotype decision making remains unknown. Recently developed methods exploring chromatin structure and transcription simultaneously will hopefully reveal this relationship in immune responses in more in detail. Similarly, live-cell imaging techniques have the potential to demonstrate the link between chromatin structures and cell fate (31, 158, 162–164).

### Immune-Mediated Disease: Rapid Response, Heterogeneity, and Non-Coding Elements and Their Interface With Genome Structure

#### Rapid Response Genes Are a Risk Factor in Potentiating Auto-Immune Disease

The structures enabling inducible gene expression which is rapid and robust, while indispensable, can cause unintended transcription at the steady state. This aberrant transcription of rapid response inflammatory genes can lead to autoimmune diseases. Specifically, cytokine genes form poised chromatin structure by trained immunity in innate immune cells and memory T cells are implicated in autoimmune disease development (165). For example, rheumatoid arthritis (RA) is mediated by cytokines released by activated macrophages and T cells contributing to damage of cartilage and bone. Synovial macrophages are derived from the embryonic yolk sac and are partially replaced by bone marrow-derived macrophages in the perinatal period. However, an increased number of bone marrow-derived synovial macrophages are associated with the development of RA (166). Specific rapid response genes, such as TNFa and IL8, are constitutively expressed by macrophages in RA patients (167, 168). Additionally, activated Th17 cells infiltrate the synovium and express TNF (169). In these cells, genomic structures are relevant to our understanding of diseases such as RA.

Aberrant rapid response gene expression patterns are in part due to alterations in local genome structure. For example, the promoter of TBX5, a TF responsible for IL8 induction, displays H3K4me3 enrichment and histone acetylation in cells derived from RA patients, leading to the overexpression of TBX5 in fibroblasts (170). Overexpressed IL8 recruits neutrophils, a major source of cartilage-degrading enzymes. In addition to local genome structures, long-range contacts are also implicated in inducing aberrant expression in genes responsible for autoimmune conditions (171). For example, risk variants for RA are located between the gene bodies of TNFAIP3 and OLIG3. Interestingly, these variants physically interact with TNFAIP3 and other immune genes - IL20RA and IFNGR1 - in B and T cells derived from RA patients, but not with OLIG3. This is despite OLIG3 being the most proximal and, therefore, most likely to interact (172). The allele containing the risk variant rs6927172 causes increased contact between the enhancer and IL20RA, resulting in greater expression in T cells. It remains unknown how exactly this variant increases interaction frequency. IL20RA, a receptor of the IL20 family proteins including IL19, IL20 and IL24, activates the JAK-STAT pathway by binding with IL20 and IL24 (173). As the JAK-STAT pathway regulates cytokine gene expression, constitutive activation of JAK-STAT induces cytokine expression, leading to the recruitment of other immune cells in this inflammatory milieu. This, in turn, leads to persistent joint inflammation, proliferative synovitis and, ultimately, damage of the underlying cartilage. Further, it is known that cytokines which display super-enhancer architecture are sensitive to JAK inhibitors (174). These results indicate that local and long-range genomic structure influence autoimmune diseases development and attenuating these aberrant responses mitigate the risks inferred.

#### Alterations in Genome Structure in Tissue-Resident Immune Cells Are a Risk Factor for Autoimmune Disease

Epigenetic reprogramming at regulatory elements of immune genes, especially rapid response genes in tissue-resident immune cells, comes at the risk of precipitating autoimmune diseases. Because tissue-resident immune cells proliferate by self-renewal once their epigenome is reprogrammed, this can have lifelong transcriptional effects for the cell. Multiple sclerosis (MS) is an important example of an autoimmune disease caused by abnormal activation of tissue-resident immune cells. MS, a neuroinflammatory disease, is characterized by chronic demyelination, oligodendrocyte death, and axonal and neuronal loss (175). Importantly, neurons in the central nervous system appear to be affected in regions of activated microglia. There are over 200 identified risk variants for MS over-and-above MHC-II and these are enriched in brain-resident immune cells, especially microglia (176). This suggests that microglia are a major contributor to the pathophysiology of MS. As in RA, MS risk variants may impact chromatin looping events. Variants located in the intron of CLEC16A interact with DEXI and up-regulate the expression of DEXI in monocytes. It remains unknown how DEXI induces MS. However, DEXI is a gene known to be responsive to dexamethasone, a common treatment for inflammatory disorders, and DEXI is likely involved in the regulation of inflammation (177).

Beyond the classic examples of RA and MS, a greater array of diseases are recognized as autoimmune. Diabetes, for example, although regarded as a metabolic disorder, is known to be fueled by the activation of adipose-resident macrophages (178). Again, risk variants associated with the development of Diabetes Mellitus Type 1 have a hand in changing the configuration of chromatin structure (179). Alzheimer’s Disease (AD) is another disease increasingly classified as auto-immune (180). Risk variants for AD are detected in microglia-specific enhancers but not in neuron- or astrocyte-specific enhancers; suggesting that aberrant activation of microglia could be responsible for AD development (181). A conceptual shift is needed to fully appreciate the causative role of inappropriate immune activation - both innate and adaptive - in disease development. Specifically, investigating chromatin structure’s role in immune regulation may reveal a range of previously-overlooked mediators of disease and therapeutic avenues that remain to be exploited.

#### The Contribution of Non-Coding Elements to Genome Architecture in Autoimmunity

Numerous studies have identified similar risk variants for autoimmune disease, nearly 90% of which are located in non-coding regions with 60% of these located within enhancers. This suggests that autoimmune diseases can be caused by mis-regulation of gene expression in key immune genes, as opposed to alterations in amino acid sequence (101, 182). Indeed, chromatin conformation investigations have shown that enhancers with risk variants interact with responsible immune genes (101, 132). Of note, it is often assumed that enhancers regulate the nearest genes; yet only 14% of enhancers with risk variants are known to do so, demonstrating the importance of considering long-range genome structure in autoimmune pathophysiology (132). The inference of the variants is complex and the mechanisms by which they influence disease development incompletely described. One possibility is that risk variants change the binding affinity of TFs. For example, the Crohn’s Disease risk variant rs17293632 directly disrupts the AP-1 motif, dysregulating the TGF-β–SMAD3 pathway (182). However, only 10-20% of risk variants are known to directly alter recognizable TF binding motifs (182). Although mutations in flanking regions also alter TF binding affinity, it remains unknown the extent to which this may impact TF binding affinity (183). Interestingly, strain-specific TF binding patterns cannot be explained by mutations (184). Likewise, several studies raise important questions about the effect of mutations on the dynamics of TF binding (185). Another mechanism by which risk variants may cause disease is by means of structural change in the genome. As shown in by the example of risk variant rs6927172 in T cells, there is no doubt that long-range genome structures are intimately involved in the causation of autoimmune disease. In addition, local structures also appears to be regulated by risk variants and a single variant is sufficient to alter chromatin accessibility (186). In this scenario, an important question is how these structural changes are controlled. One potential is the influence of TF binding: it is possible that DNA-DNA interactions are not only mediated by architectural protein such as CTCF, but also by an array of TFs through phase separation. A notable example of the influence of risk variants on loop structure is asthma risk variant which affect CTCF mediated loops and regulate ORMDL3 gene, a negative regulator of IL2 in CD4+ T cells (187). However, as above, this may not be sufficient to explain all cases. Another possibility is the role of certain lncRNAs, such as eRNAs. Some lncRNAs are known to mediate enhancer-promoter interactions (188), likely *via* their secondary structures and protein binding potential (189). Indeed, the strong linkage disequilibrium between certain risk variants and lncRNA loci are implicated in changes to lncRNA structure (190). For example, it is predicted that rs11153299 and rs2038013, which have a strong linkage disequilibrium with inflammatory bowel disease risk variant, disrupt the structure of lncRNA TRAF3IP2-AS1. Mutations, therefore, may either lead to lncRNA expression suppression or deformation of their secondary structures. Further studies are needed to begin interrogating these unknowns.

#### Epigenetic Modification as a Precipitant of Autoimmunity in the Context of Genome Architecture

As discussed above, in at least some cases, chromatin structure is implicated in autoimmune disease development. However, alteration of chromatin structure by genetic variants alone is insufficient to precipitate autoimmunity. Most autoimmune conditions occur in older individuals with younger individuals never being risk-variant carriers. HSCs may acquire critical causative mutations only late in life (191). However, we assume this to be a limited driver of autoimmunity as these mutations occur randomly and cannot explain familial patterns of autoimmune disease. To explain this, we propose a model in which altered chromatin structure caused by genetic variants creates a scaffold upon which age-induced epigenetic changes induce abnormal inflammation. For example, the histone deacetylation activity of Sirtuin family proteins, such as SIRT1, declines with age (192). As these senescent cells accumulate histone acetylation marks, enhancers become activated. In cells which are risk-variant carriers, chromatin loops mediated by these variants bridge activated enhancers and inflammatory gene promoters resulting in aberrant expression. As rapid response genes form poised structures in the naïve state, these genes are activated easily in older people carrying risk variants. Techniques able to capture enhancer-promoter interaction and histone modification simultaneously - such as HiChIP and ChIA-Drop - will be powerful tools in fully understanding autoimmune disease development (36, 193). Interestingly, histone modification variations between individuals and single cells increase with age, suggesting that heterogeneity of the chromatin structure may play a role in autoimmune disease development (194). Single cell chromatin profiling techniques will be a key to understand autoimmune disease development as well.

## Discussion

We reviewed that immune cells make rapid response possible by preformed “ready-to-respond” and it’s a risk of autoimmune diseases. Also, we argued that genome structure is heterogenous and how it possibly affects to immune gene expression and decision of phenotype after activation. Still, it is largely unknown the role of heterogeneity of the genome structure in immune response. For example, is plasticity of genome structure important for response to diverse pathogens? Also, it is unknown whether or not there is interrelationship between lower scale genome structure such as nucleosome and higher scale such as DNA looping for heterogeneity. As some TFs mediate DNA loops and depletion of nucleosome is essential for TF binding, DNA looping and nucleosome position will show interrelationship and are not completely independent (195). The techniques that detect multiple data simultaneously such as transcription, nucleosome and DNA loops and live-cell imaging of epigenome will bring a novel insight to these questions.

## Author Contributions

LD, MM, and YN conceived, designed and wrote a paper. All authors contributed to the article and approved the submitted version.

## Conflict of Interest

The authors declare that the research was conducted in the absence of any commercial or financial relationships that could be construed as a potential conflict of interest.
